# Comparing strategies for matching mortality forecasts to the most recently observed data: exploring the trade-off between accuracy and robustness

**DOI:** 10.1186/s41118-018-0041-y

**Published:** 2018-09-29

**Authors:** Lenny Stoeldraijer, Coen van Duin, Leo van Wissen, Fanny Janssen

**Affiliations:** 1 Statistics Netherlands, Henri Faasdreef 312, PO Box 24500, 2492 JP Den Haag, The Netherlands; 2 Population Research Centre, University of Groningen, PO Box 72, 9700 AB Groningen, The Netherlands; 3 Netherlands Interdisciplinary Demographic Institute, Lange Houtstraat 19, 2511 CV Den Haag, The Netherlands

**Keywords:** Mortality forecasting, Robustness, Accuracy, Jump-off rates

## Abstract

**Background:**

Given the increased link between retirement age and payments to the development in life expectancy, a precise and regular forecast of life expectancy is of utmost importance. The choice of the jump-off rates, i.e. the rates in the last year of the fitting period, is essential for matching mortality forecasts to the most recently observed data. A general approach to the choice of the jump-off rates is currently lacking.

**Objective:**

We evaluate six different options for the jump-off rates and examine their effects on the robustness and accuracy of the mortality forecast.

**Data and methods:**

Death and exposure numbers by age for eight European countries over the years 1960–2014 were obtained from the Human Mortality Database. We examined the use of model values as jump-off rates versus observed values in the last year or averaged over the last couple of years. The future life expectancy at age 65 is calculated for different fitting periods and jump-off rates using the Lee-Carter model and examined on accuracy (mean absolute forecast error) and robustness (standard deviation of the change in projected e65).

**Results:**

The choice for the jump-off rates clearly influences the accuracy and robustness of the mortality forecast, albeit in different ways. For most countries using the last observed values as jump-off rates resulted in the most accurate method, which relates to the relatively high estimation error of the model in recent years. The most robust method is obtained by using an average of observed years as jump-off rates. The more years that are averaged, the better the robustness, but accuracy decreases with more years averaged.

**Conclusion:**

Carefully considering the best choice for the jump-off rates is essential when forecasting mortality. The best strategy for matching mortality forecasts to the most recently observed data depends on the goal of the forecast, the country-specific past mortality trends observed, and the model fit.

## Introduction

The growth in public expenditure, such as expenditure on state pension, due to an ageing population is one of the key challenges in European countries (Lanzieri 2011). To ensure the sustainability of the pension system expenditures, pension reforms in several countries in Western Europe have been carried out, linking the retirement age and/or retirement payments to the rapidly increasing life expectancy (Carone et al. 2016). In some countries, such as Finland and the Netherlands, the link is made with a forecasted remaining life expectancy (OECD 2015). Given the increased link between retirement age and/or retirement payments to the development in life expectancy, a precise and regular forecast of life expectancy is of utmost importance.

The growing relevance of life expectancy forecasts has resulted in a lot of attention regarding the quality of mortality forecasts. There has been a growing range of models for forecasting mortality and studies performing quantitative and qualitative comparisons of these models (Booth and Tickle 2008; Cairns et al. 2011; etc.). Also, in recent literature, there has been a lot of attention for the elements that influence the quality of mortality forecasts, i.e. the fitting period (Booth et al. 2002) or additional information, such as smoking (Janssen et al. 2013) or trends in other countries (Li and Lee 2005). Less attention has been given, however, to the choice of the jump-off rates, i.e. the rates in the last year of the fitting period or jump-off year (Booth et al. 2006). The choice of the jump-off rates is leading when matching the mortality forecast to the most recently observed data. The matching is in turn important for a precise and regular forecast of the life expectancy and thus for the determination of the retirement age and payments.

The choice of the jump-off rates is essential for matching mortality forecasts to the most recently observed data (Lee and Miller 2001; Booth et al. 2006) and is a practical consideration in every mortality forecast, regardless of the method chosen. A different choice of the jump-off rates may improve the accuracy of a single forecast and/or reduce the discontinuity between the last observed death rate and the first forecasted death rate (Lee and Miller 2001; Booth et al. 2006). A forecast is called accurate if the out-of-sample forecast errors, examined using historical data, are small (Booth et al. 2008). An accurate method produces precise forecasts which are relevant to determine the retirement age in a future year based on the forecasted life expectancy. However, the choice of the jump-off rates can also influence how much successive forecasts differ, thereby affecting the robustness of the forecast (Cairns et al. 2011). A forecast is called robust if only modest changes in the forecasts occur after a small change to the sample period (for example, adding the latest mortality data). For instance, if a retirement age in a future year is set based on the forecasted life expectancy, it is undesirable if a forecast based on one more year of data gave a different outcome. Both accuracy and robustness are important for a mortality forecast (Cairns et al. 2011) but can be differently affected by the choice of the jump-off rates.

The choice for the jump-off rates being more a practical problem than a theoretical one is also highlighted by the fact that there are only four papers about the choice for the jump-off rates. Lee and Carter (1992) used model values (i.e. fitted rates in the jump-off year) as jump-off rates and accepted the discontinuity in observed to forecasted death rates. They stated that the jump-off bias affects only death rates which are absolutely very low and have little impact on the forecasted life expectancy. However, Bell (1997) as well as Lee and Miller (2001) concluded that a correction for the jump-off bias improves the accuracy of the forecast of life expectancy, especially in the early years of the forecast. They used the last observed values (i.e. actual rates) as jump-off rates. Finally, Booth et al. (2006) evaluated as well a 2-year average of the last observed values as jump-off rates as part of the evaluations of Lee-Carter models and variants. The literature thus gives us only three options to choose from: model values, last observed values, and a 2-year average of last observed values.

In practice, statistical and actuarial offices use different options for the jump-off rates (mostly last observed values) and, with a new update of the forecast, the choice of the jump-off rates might differ as well. Often, however, it is not explained how they reached these jump-off rates. There are some examples where there are more extensive adjustments of the jump-off bias, but they are relevant for the practical problem at hand and not for universal use (for instance, the statistical office of New Zealand adjusts the rates in the first few years to give plausible life expectancy at birth and death numbers (Woods and Dunstan 2014). In fact, a general approach on how to choose between different options for the jump-off rates seems to be lacking.

In the literature (Lee and Carter 1992; Bell 1997; Lee and Miller 2001) and in practice, the jump-off rates are adjusted to improve the accuracy of the forecast. Also, quantitative and qualitative comparisons of different models are mainly focused on improving accuracy. However, in light of regular forecasts for the determination of retirement age and payments, it is also of interest to take into account the robustness of the method.

This article examines the effects of different options for the jump-off rates on the accuracy and the robustness of the mortality forecast. This information can be used to determine the optimal choice for a given forecast, which will depend on the relative importance of accuracy and robustness for the applications for which the forecast is used.

We will do so by forecasting future life expectancy at age 65 for eight Western European countries using different fitting periods and six different options for the jump-off rates. An accurate and robust forecast of the life expectancy at age 65, with the mortality forecast matched as optimally as possible to the most recently observed data, is important for the pension reforms in Western Europe.

## Data and methods

### Data

For the analysis, deaths and exposures by calendar year and single year of age from the Human Mortality Database (2018) are used, from 1960 to 2014. In our calculations, we aggregated the data for ages 95 and over (Wunsch and Termote 1978).

To contribute to the debate about the retirement age in Western Europe, and to observe commonalities and differences in the effect of the choice of the jump-off rates for the mortality forecast, data from eight Western European countries is used: the Netherlands (NLD), France (FRA), Belgium (BEL), Spain (ESP), Finland (FIN), United Kingdom (UK), Norway (NOR), and Sweden (SWE).

These countries experienced foremost fairly regular mortality trends in the chosen period, for which extrapolative forecasting methods would be suitable. Differences exist however in the extent of mortality decline between the individual countries.

We selected data from 1960 up until 2014, which gave us the opportunity to compute forecasts for the more recent years in the period in order to test the accuracy of the forecasting method.

### Model

Many statistical offices are currently using extrapolation methods to forecast mortality (Stoeldraijer et al. 2013). To evaluate the effect of different choices for the jump-off rates, we will apply the most used extrapolation method: the Lee-Carter method (Lee and Carter 1992; Booth and Tickle 2008).

The Lee-Carter model (Lee and Carter 1992) is given by:

$$ \ln \left({m}_{x,t}\right)={a}_x+{b}_x{k}_t+{\varepsilon}_{x,t} $$

where *m*_*x*, *t*_ denotes the death rate at age *x* and year *t*, *a*_*x*_ equals the average over time of ln(*m*_*x*, *t*_), *b*_*x*_ is the set of age-specific constants that describe the relative rate of change at any age, *k*_*t*_ denotes the underlying time development and *ε*_*x*, *t*_ the residual error (Lee and Carter 1992). Singular Value Decomposition is used to estimate *b*_*x*_ and *k*_*t*_ under the assumptions $\sum \limits_x{b}_x=1$ and $\sum \limits_t{k}_t=0$ (Lee and Carter 1992). After estimation, *k*_*t*_ is extrapolated using a random walk with drift (as also found by Lee and Carter 1992, after carrying out the standard model specifications (see Box and Jenkins 1970)).

### Jump-off rates

For the analysis, three options for the jump-off rates are compared:

- Jump-off rates equal to the model values in the last year of the fitting period (Lee and Carter 1992);
- Jump-off rates equal to the last observed death rates (Lee and Miller 2001); this corresponds to taking *a*_*x*_ equal to the last observed values of ln(*m*_*x*, *t*_) and *k*_*t*_ equal to zero in the last observed year;
- Jump-off rates equal to an average of multiple years of the observed death rates; this corresponds to taking *a*_*x*_ equal to the average of multiple observed years of ln(*m*_*x*, *t*_) and ${k}_{\sum {t}_i/n}$, the midpoint of the years on which is averaged, equal to zero.

By distinguishing four alternatives for the last option (average over 2, 3, 4, or 5 years) we end up with in total six different alternatives.

### Analysis

For the analysis, we made for each country ten forecasts of life expectancy at age 65, men and women combined, using data for ten different fitting periods: from 1960 to 2005, 1960 to 2006, …, and 1960 to 2014. The forecasts are calculated using the six different alternatives of the jump-off rates: the model values, the observed values, and an average of two/three/four/five observed values.

Subsequently, we compared between the different choices for the jump-off rates the accuracy (fit of the model) and the robustness of the forecast, as these are the most important evaluation criteria for mortality forecasts (e.g. Dowd et al. 2010a, 2010b; Cairns et al. 2011; Booth and Tickle 2008).

A model is accurate if the out-of-sample forecast errors, examined using historical data, are small (Booth et al. 2008). For evaluating the accuracy we used the mean absolute forecast error (MAFE) (Booth et al. 2006). The MAFE measures how close forecasts are to the eventual outcomes. The smaller the error, the more accurate the forecast, given the option for the jump-off rates. For each country and choice of the jump-off rates, we calculated the MAFE by comparing the forecasted values with the actual values of the life expectancy at age 65. The MAFE for the first year of the forecasting period (i.e. the first year after the fitting period) was calculated using fitting periods 1960–2005, …, 1960–2013. The forecast of 2006 (with fitting period 1960–2005) was compared with the actual value in 2006, the forecast of 2007 (with fitting period 1960–2006) was compared to the actual value in 2007, and in a similar way for the subsequent forecasts until the forecast of 2014 (with fitting period 1960–2013) which is compared to the actual value of 2014. The errors are then averaged across the nine different forecasts (2006–2014). The MAFE for the second year of the forecasting period was calculated using fitting periods 1960–2005, …, 1960–2012 and then averaged across the eight different forecasts. In the results (see Table 1), only the MAFE for the first and fifth year of the forecasting period are presented, because the results for the intervening years did not provide useful additional information.

**Table 1:** Mean absolute forecast error (MAFE) of remaining life expectancy at age 65 for the first and fifth year in the forecasting period, for six different choices of the jump-off rates applied to a Lee-Carter model, for eight Western European countries, men and women combined, fitting periods 1960–2005, 1960–2006, …, 1960–2014

Jump-off rates	FRA	ESP	SWE	BEL	UK	NOR	FIN	NLD	Av^1^
Mean absolute forecast error in the first year of the forecasting period									
Model values	0.16	0.16	*0.24*	**0.10**	*0.56*	*0.24*	*0.41*	*0.39*	*0.28*
Last observed values	**0.13**	0.17	0.08	0.14	**0.13**	0.12	0.10	**0.13**	**0.12**
Average 2 years observed	0.13	**0.15**	**0.07**	0.15	0.16	**0.11**	**0.08**	0.16	0.13
Average 3 years observed	0.13	0.16	0.09	0.14	0.19	0.12	0.09	0.21	0.14
Average 4 years observed	0.14	0.17	0.10	0.15	0.24	0.13	0.12	0.26	0.16
Average 5 years observed	*0.16*	*0.19*	0.11	*0.17*	0.28	0.16	0.16	0.32	0.19
Mean absolute forecast error in the fifth year of the forecasting period									
Model values	0.20	**0.17**	*0.31*	0.18	*0.76*	*0.45*	*0.41*	*0.55*	*0.38*
Last observed values	**0.16**	0.30	**0.10**	**0.17**	**0.38**	**0.20**	**0.13**	**0.31**	**0.22**
Average 2 years observed	0.17	0.32	0.11	0.18	0.41	0.21	0.14	0.35	0.24
Average 3 years observed	0.18	0.35	0.13	0.19	0.44	0.24	0.15	0.41	0.26
Average 4 years observed	0.20	0.38	0.16	0.20	0.46	0.29	0.18	0.47	0.29
Average 5 years observed	*0.21*	*0.40*	0.18	*0.21*	0.49	0.34	0.19	0.55	0.32

The lowest MAFE is marked in bold, the highest MAFE in italics

^1^Unweigthed average of all eight countries

Furthermore, to explain the results regarding the accuracy of the forecast (fit of the model), we calculated the mean absolute (percent) error over the period 1960–2014 and the mean error over the period 2005–2014 of the log death rates, limited to age 65 and above, of the Lee-Carter forecast (estimated over the period 1960–2014).

With a robust forecast, only modest changes would occur to the forecasted life expectancy after a small change to the sample period (e.g. adding one more year) (Cairns et al. 2011). It is important here to look at the stability of each incremental change to the sample period. This is relevant in the case a forecast is regularly updated, i.e. when a new forecast is made each time new data becomes available. Normally, robustness is measured by looking at the changes in model parameters (Cairns et al. 2008). However, these parameters do not depend on the option that is used for the jump-off rates. Therefore, to evaluate the robustness of the forecast given the different options for the jump-off rates, we calculated the standard deviation (SD) of the increase/decrease of the (out-of-sample) life expectancy at age 65 in 2020 obtained for ten successive forecasts using fitting periods 1960–2005, …, 1960–2014. A lower SD means that the forecast is more robust.

## Results

### Past trends in remaining life expectancy at age 65

**Fig. 1:**
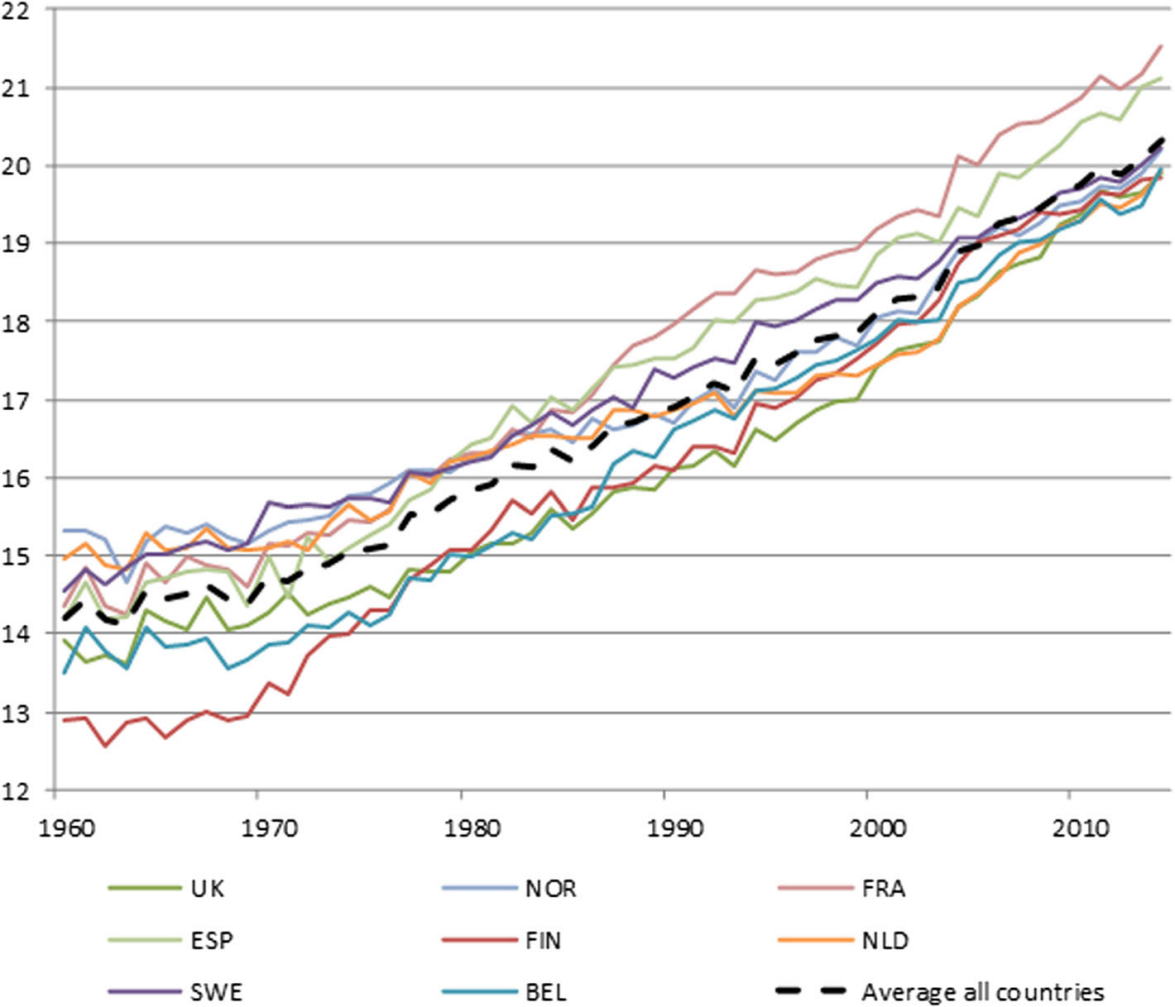
Life expectancy at age 65, 1960–2014 for eight countries, men and women combined

Over the period 1960 to 2014, the remaining life expectancy at age 65 (e65) increased in the eight selected European countries, for men and women combined (Fig. 1). On average, from 14.2 years in 1960 to 20.3 years in 2014, France has seen the largest increase over the whole period, while Norway has seen the lowest increase. Especially for the Netherlands and the UK, there was a higher increase in e65 in the last decade of the observation period than in the decades before. In 2014, the highest e65 was observed for France (21.5 years) and the lowest for Finland (19.8 years), the UK(19.9 years), and the Netherlands (19.9 years).

### Effect of choice of the jump-off rate on the accuracy of mortality forecast

The six different options for the jump-off rates resulted in clear differences in the outcome for the accuracy of the forecast (see Table 1): on average, there was a difference of 0.18 between the option which gave the minimum accuracy and the option which gave the maximum accuracy for the first year of the forecasting period. The minimum difference was found for France (0.03) and the maximum difference was found for the UK (0.43). With a large difference, such as for the UK, it makes a clear difference which option is chosen for the jump-off rates. The larger the difference between the options for the jump-off rates, the more important it is to choose the correct jump-off rates so that the accuracy of the forecast can be improved.

Using the last observed values as jump-off rates or an average of 2 years resulted in the most accurate forecast in the first year of the forecasting period for most countries (looking at the minimum MAFE by country). Only for Belgium, the most accurate forecast was achieved by using the model values as jump-off rates. The minimum MAFE in the first year of the forecast ranged from 0.07 (Sweden) to 0.15 (Spain). The most accurate forecast for the fifth year of the forecasting period was achieved by using the last observed values as jump-off rates, except for Spain, where the most accurate forecast was achieved by using the model values as jump-off rates. The minimum MAFE in the fifth year of the forecast ranged from 0.10 (Sweden) to 0.38 (the UK).

Except for France, Spain, and Belgium, using the model values as jump-off rates resulted in the least accurate forecast in the first year. For France, Spain, and Belgium, using the average of five observed years resulted in the least accurate forecast in the first year. The least accurate for the fifth year of the forecasting period showed the same pattern as the accuracy for the first year of the forecasting period. For all countries, the accuracy decreases distinctly with the averaging of more years.

Generally, the MAFE in the fifth year is higher than in the first year (using the same option for the jump-off rates), reflecting that uncertainty further in the future is greater.

The most optimal choice for the jump-off rates for an accurate forecast is related to the error the model makes in the recent estimation period (fitting errors, Table 2). The mean error over the estimation period 2005–2014 is close to zero for Belgium. This was the only country for which the model values as jump-off rates gave the most accurate results. For Sweden, the UK, Norway, Finland, and the Netherlands, the mean error is negative, i.e. the recent period was underestimated by the Lee-Carter model. For these countries, the model values as jump-off rates were the worst option for an accurate forecast. These countries had a stronger increase in e65 in the recent decade compared to earlier decades. Hence, using observed values as jump-off rates would mean the forecast is already closer to the observed future values than using the model values. For France and Spain, the mean error was positive and differences in accuracy between the options for the jump-off rates were small.

**Table 2:** Mean absolute (percent) error over the period 1960–2014 and mean error over the period 2005–2014 of the log death rates limited to age 65 and above of the Lee-Carter forecast estimated over the period 1960–2014, for eight Western European countries, men and women combined

	FRA	ESP	SWE	BEL	UK	NOR	FIN	NLD
Mean absolute error (1960–2014)	0.028	0.040	0.031	0.032	0.036	0.037	0.046	0.036
Mean absolute percent error (1960–2014)	1.20	1.72	1.49	1.61	1.51	1.61	2.34	1.65
Mean error (2005–2014)	0.017	0.019	− 0.016	0.001	− 0.035	− 0.016	− 0.028	− 0.019

### Effect of choice of the jump-off rate on the robustness of mortality forecast

The choice of the jump-off rates clearly affected the robustness of the forecast: on average, there was a difference of 0.18 between the minimum value of the SD and the maximum value of the SD. The minimum difference was found for the Netherlands (0.10) and maximum for Finland (0.36) (Table 3).

**Table 3:** Standard deviation (SD) of the increase/decrease of the life expectancy at age 65 in 2020 between ten successive forecasts (fitting periods 1960–2005, 1960–2006, …, 1960–2014) for six different choices of the jump-off rates applied to a Lee-Carter model, for eight Western European countries, men and women combined

Jump-off rates	FRA	ESP	SWE	BEL	UK	NOR	FIN	NLD	Av^1^
Standard deviation in 2020									
Model values	0.11	0.11	*0.23*	0.17	0.15	*0.26*	*0.51*	*0.17*	*0.22*
Last observed values	*0.19*	*0.24*	0.09	*0.20*	*0.17*	0.12	0.16	0.13	0.16
Average 2 years observed	0.11	0.10	0.06	0.10	0.11	0.09	**0.15**	0.08	0.10
Average 3 years observed	0.10	0.09	0.06	0.08	0.09	0.09	0.18	0.08	0.10
Average 4 years observed	0.08	**0.05**	**0.05**	0.06	0.07	0.10	0.18	0.08	0.08
Average 5 years observed	**0.07**	0.05	0.06	**0.06**	**0.06**	**0.09**	0.16	**0.07**	**0.08**

The lowest SD is marked in bold, the highest SD in italics

^1^Unweigthed average of all eight countries

Using an average of recent observed years as jump-off rates results in a lower standard deviation of the increase/decrease of the life expectancy at age 65 in 2020 and thus a more robust method. This holds for all countries under study. The minimum standard deviation (per country) ranges from 0.05 (Spain and Sweden) to 0.15 (Finland). For most countries, an average of at least 4 years gives the minimum standard deviation. The difference between a 2-year average and a 5-year average (maximum 0.5) is small compared to the differences with last observed or model values.

The worst options for the jump-off rates in terms of the robustness are either the model values (Sweden, Norway, Finland, and the Netherlands) or the observed values (France, Spain, Belgium, and the UK). The maximum standard deviation (per country) ranges from 0.17 (the UK and the Netherlands) to 0.51 (Finland).

The fact that the last observed values as jump-off rates are not performing well on robustness is related to the nature of the data: the observed life expectancy fluctuates greatly from year to year. By using the last observed values as jump-off rates in the forecasting model, also the future values will fluctuate when recent data is added. Taking an average of multiple years makes sure there are fewer fluctuations. The model values are similar to taking an average, but over the whole period in that case. Because the relative decline of the model will influence the forecast more when using the model values as jump-off rates than an average of recent observed years, the robustness of the forecast is better using the average as jump-off rates.

Another feature of the results is also apparent: the countries in the south of Western Europe (France and Spain) have the last observed values as the worst option for the jump-off rates, but for the countries in the north of Western Europe (Sweden, Norway, and Finland) have the model values as the worst option. For France and Spain, the model values are not much different with the average as jump-off rates, while for Sweden, Norway, and Finland, the difference between the last observed values and the average are small. Belgium, the UK, and the Netherlands are more in between (in location and in the results). For these three countries, it also holds that the difference between using the model values and last observed values as jump-off rates does not differ as much as for the other five countries.

## Discussion

We evaluated the accuracy and robustness of the forecast of life expectancy at age 65 in Western Europe for six different options for the jump-off rates. We observed that the options for the jump-off rates clearly influence the accuracy and robustness of the mortality forecast, albeit in different ways. For most countries, the most accurate forecast resulted from taking the last observed values as jump-off rates, which relates to the relatively high estimation error of the model in recent years. The most robust forecast was obtained by using an average of the most recent observed years as jump-off rates. The more years that are averaged, the better the robustness, but accuracy decreases with more years averaged. The best choice for the jump-off rates, thus, seems to depend on whether you are interested mainly in accuracy or robustness, on the country-specific past mortality trends, or the model fit.

**Fig. 2:**
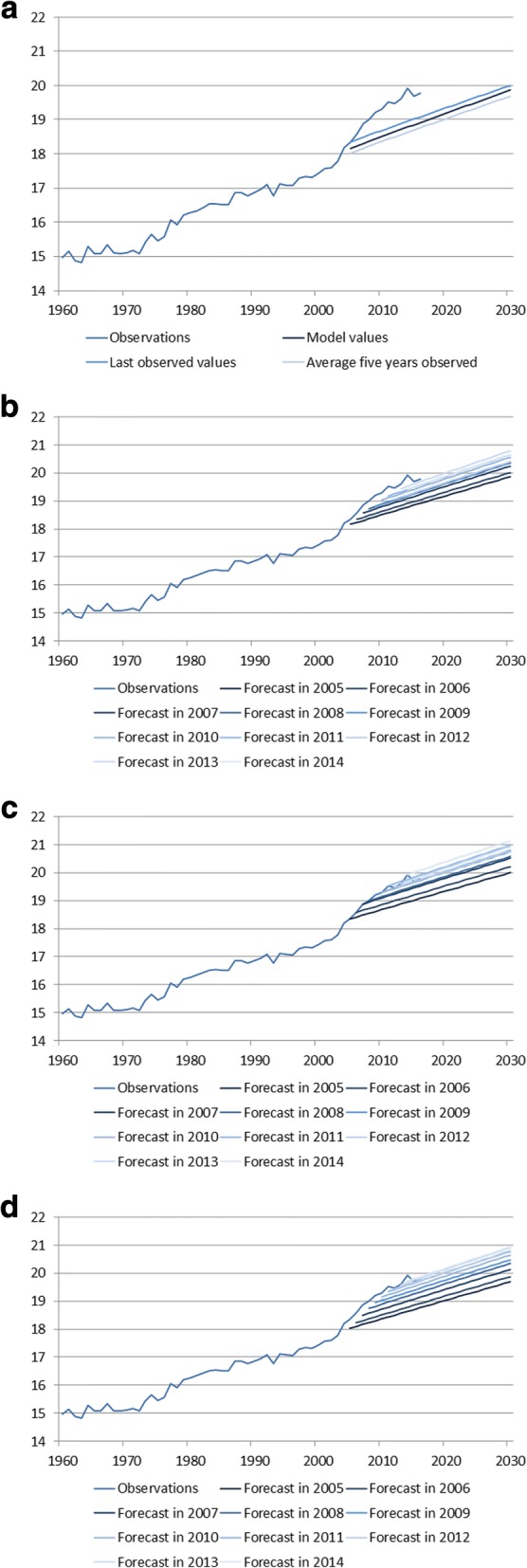
Example of forecasts with different choices for the jump-off rates, the Netherlands, men and women combined, Lee-Carter model, fitting periods 1960–2005, 1960–2006, …, 1960–2014. **a** Forecast in 2005 with three options of the jump-off rates. **b** Forecasts with jump-off rates equal to model values. **c** Forecasts with jump-off rates equal to last observed values. **d** Forecasts with jump-off rates equal to the average 5 years observed

The influence of the choice of the jump-off rates on the accuracy and robustness of the forecast can be substantial. Figure 2 in Appendix 1 gives an example for the Netherlands of a forecast with the model values as jump-off rates, a forecast with the last observed values as jump-off rates, and a forecast with an average of five observed years as jump-off rates, with different fitting periods. The forecasts with the model values as jump-off rates are not accurate, i.e. there are large gaps between the observed values and the forecasts in the first year. The forecasts with the model values are also not robust: the successive forecasts, using different fitting periods, show large differences (i.e. increases and decreases between successive forecasts) between the successive forecasted e65 for a particular year. For the forecasts with the last observed values as jump-off rates the accuracy is improved, and, from the analysis, the most accurate from the six options for the jump-off rates. However, the successive forecasts are also showing large differences between the successive forecasted e65 in a particular year. Lastly, the successive forecasts with an average of five observed years as jump-off rates are slowly increasing with each new year of data added to the fitting period. This option for the jump-off rates was the most robust for the Netherlands.

### Evaluation of analysis

We assessed the effect of the choice of the jump-off rates by means of two important evaluation criteria for a mortality forecasting method: robustness and accuracy (Dowd et al. 2010a, 2010b; Cairns et al. 2011). A third evaluation criterion for evaluating a mortality forecast is plausibility (Cairns et al. 2011): is the outcome of the forecast reasonable given what we know? This is rather a subjective issue for which there are no objective measures, and for that reason, we did not include it in the analysis. Nonetheless, plausibility is important to consider when performing a mortality forecast. A plausible future age pattern is an important issue related to the plausibility of the results. Different characteristics of the jump-off rates, such as a rough age pattern of the last observed values, have an effect on the plausibility of the future age pattern of mortality. To limit the effect of the choice of the jump-off rates on the plausibility of the future age pattern, smoothing the observed mortality rates by age is recommended.

We performed the different mortality forecasts using the Lee-Carter method, which is frequently used for mortality forecasting in practice (Stoeldraijer et al. 2013), as benchmark method (Booth and Tickle 2008), and as the basis for more recent mortality forecasting models (Booth and Tickle 2008; Lee and Carter 1992). The Lee-Carter method, however, is known to be biased and tends to underpredict future mortality (Bell 1997; Lee 2000; Lee and Miller 2001; Booth et al. 2002; Girosi and King 2007; Liu and Yu 2011), as we have also seen in Table 2 where the mean error in the last ten years of the fitting period was negative for most countries. Therefore, differences between the last observed values and the model values tend to be relatively large. For this reason, we performed a sensitivity analysis using two additional models: (i) a Lee-Carter model using three principal components (Appendix 2), because based on earlier research, it is unnecessary to adjust the jump-off rates when several principal components are used (Hyndman et al. 2013); and (ii) the Cairns-Blake-Dowd model (Cairns et al. 2006; Appendix 3), which is considered a different stochastic model compared to the Lee-Carter model and widely used in actuarial sciences. The results show smaller differences in outcomes compared to differences we observed earlier with the Lee-Carter model, but, especially for accuracy, the importance of the jump-off rates remains. This highlights the importance of the model for the best choice of the jump-off rates.

We showed the results of our analysis for men and women combined. Similar results are observed however for men and women separately (see Tables 8 and 9 in Appendix 4). Also for men (with the exception of Finland) and women separately, an average of multiple years as jump-off rates was preferred for the most robust forecast. For the most accurate forecast, there was some more variation in the results for men and women separately compared to men and women combined. For men in France and Spain, the forecast is most accurate when using model values as jump-off rates, although accuracy is only slightly higher compared to the last observed values. For women, the most accurate forecast in the fifth year of the forecasting period is obtained by using the last observed values. The accuracy of the forecast for the first year of the forecasting period shows for women mostly small differences between choices for the jump-off rates but resulted in model values (France, Sweden), last observed values (Belgium, the UK), and an average (Spain, Norway, Finland, the Netherlands).

We deliberately computed the accuracy and the robustness measures directly for life expectancy at age 65, because of the use of this indicator in the pension reforms. For different contexts, e.g. life insurance and pension valuation, an evaluation of other outcomes (e.g. death rates or probabilities) would be relevant and could lead to different outcomes. That is, for different age groups the model fit, and subsequently, the choice of the jump-off rates might be different. Booth et al. (2006) compared both errors in life expectancy and log death rates when analysing the accuracy for different choices of the jump-off rate. They concluded that the accuracy in log death rates does not necessarily translate into accuracy in life expectancy. Analysis based on forecasted log death rates might therefore lead to different conclusions, but in general, last observed values as jump-off rates would give the most accurate forecast (Booth et al. 2006). The above indicates that the context of the forecast determines the outcome measure used in the analysis of the jump-off rates and, hence, the final choice for the best jump-off rates.

### Generalizability of our outcomes

We evaluated the results based on the life expectancy at age 65 in relation to pension reforms. Results based on the life expectancy at birth (e0) are very similar to the results based on e65 (Appendix 5). The differences between the six options for the jump-off rates for both accuracy and robustness are slightly larger for e0 than for e65. This means that our conclusions can be generalised to other ages of life expectancy.

We focused our analysis on Western Europe, because of the prevalence of the pension reforms. Our findings can be generalised to countries which have seen similar trends in the past. For example, the results for the Netherlands are expected to be close to the results for Denmark, since both experienced a stagnation of the increase in life expectancy at approximately the same time (Janssen et al. 2004). Similarly, our results for the remaining Western European countries can be generalised to other countries exhibiting fairly regular increases in life expectancy, like Japan since 1970 (Leon 2011). Generalising our results to Eastern Europe, however, will be more daunting because these countries experienced very different past mortality trends due to the health crisis from 1975 onwards (McKee and Shkolnikov 2001; Vallin and Meslé 2004; Leon 2011). The Lee-Carter method is most likely not suited to account for these specific past mortality trends (Bohk and Rau 2015). Before evaluating different choices for the jump-off rates in the context of Eastern Europe, first, the forecasting method needs to be improved.

### Recommendations

Following our findings, we recommend the goal of the forecast, and the related emphasis on accuracy, robustness, or both, to be leading for determining the best choice of the jump-off rates.

If the goal of the mortality forecast is focused on accuracy, it is relevant to examine the error of the estimates of the model over the period it is applied to, following its importance in explaining our results for accuracy. We recommend the model values as most suitable as jump-off rates for an accurate forecast when the errors are small. We recommend the last observed values as most suitable jump-off rates when the model errors are large and there is an underestimation of the model in the most recent period. With large errors and an overestimation of the model in the most recent period, we recommend to use the model values as jump-off rates, following our results of men and women separately.

If the goal of the mortality forecast is focused on robustness, we recommend using an average of multiple years as jump-off rates, as it was the most suitable for a robust forecast for all countries in our analysis. There was little difference in the outcomes between a 2-year average and a 5-year average; thus, the number of years used in the averaging is less important. Robustness becomes more important in situations where the forecast is made regularly, for instance when the future retirement age based on the forecasted life expectancy needs to be determined every year.

Because often the goal of the forecast is focused both on accuracy and robustness, the most optimal choice for the jump-off rates must give the most accurate as well as the most robust forecast. For each country in our analysis, there was no option of the jump-off rates that guaranteed accuracy and robustness at the same time. Thus, there always has to be a trade-off between accuracy and robustness. Therefore, we recommend looking into developing a choice for the jump-off rates that is both accurate and robust. Our four recommendations for determining the best choice for the jump-off rates that give both an accurate and robust forecast are as follows: (1) Because the accuracy of the forecast decreases distinctly with the averaging of more observed years as jump-off rates, whereas the robustness of the forecast stayed approximately the same, it is preferable to use an average using as few years as possible to improve the accuracy with a robust forecast. (2) Using the observed values instead of the model values in case the model fits the data well does not improve accuracy and deteriorates the robustness. Thus, in the case the model fits well, it is best to use the model values as jump-off rates and not the observed values as is often done by force of habit. (3) The further ahead, the less accurate the forecast gets. This means that the relative price you pay for more robustness is lower for a forecast further in the future. If the forecast further in the future is of more importance than the short-term forecast, there should be a greater value attached to the robustness of the forecast, and thus the best option for the most robust forecast can be selected. (4) In line with the previous recommendations, to best unite the results for robustness and accuracy, we would recommend interpolation (see Appendix 6 for an example). Robustness is more important for the long-term forecast (for instance, from 5 years in the future) as a result of the increasing uncertainty with duration. For the first few years, accuracy would be more relevant because data for these years will be available quickly. Our recommendation would be to start with a forecast using a jump-off rate that is the most accurate in the first year. Subsequently, make a forecast that is most robust in, say, the fifth year of the forecast period. Between the two forecast, each year, more weight should be given to the most robust forecast, i.e. we recommend interpolating from the most accurate forecast to the most robust forecast. By interpolating between the two forecasts, both accuracy in the first year of the forecast and robustness of the forecast 5 years ahead is obtained.

An additional issue to consider is to match the forecast to recent data, it is important that it is of good quality. Preliminary data might underestimate or overestimate the life expectancy. Using jump-off rates based on this data might not work well for the accuracy (to final data) of the forecast. It might also turn out to be disadvantageous for the robustness if the preliminary data is replaced by final data. The use of preliminary data is therefore not recommended when matching the forecast to recent data.

## Overall conclusion

The choice of the jump-off rates clearly influences, in different ways, the accuracy and robustness of the mortality forecast. It is therefore important to carefully consider the best choice for the jump-off rate when forecasting mortality. This is especially relevant when a forecast is regularly updated, as is the case for the pension reforms.

The best choice depends on the goal of the forecast, the country-specific past mortality trends observed, and the model fit. Because the best option of the jump-off rates for accuracy (most often last observed values) and the best option for robustness (average of observed years) are not equal, there will always have to be a trade-off between the two. The recommendations presented, of which interpolation between the jump-off rates with optimal accuracy and optimal robustness combines accuracy and robustness, give guidelines to make a just trade-off between accuracy and robustness of the forecast.

## Figures and Tables

**Fig. 3:**
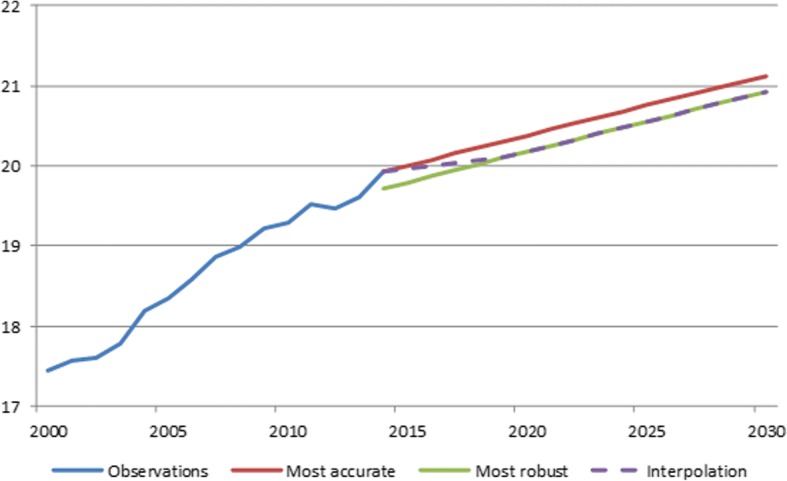
Example of forecasts with interpolation from the forecast with the option for the jump-off rates which is considered the most accurate to the forecast with the option for the jump-off rates which is considered the most robust, the Netherlands, men and women combined, Lee-Carter model, fitting period 1960–2014

## Appendix 2

In Tables 4 and 5, the results of the different choices for the jump-off rates with a Lee-Carter model using three principal components are presented. The different model mostly influences the robustness results, with for France, Spain, and the Netherlands, the model values as jump-off rates as the most optimal. Differences in outcome between the choices of the jump-off rates are much smaller than the difference in outcome with the Lee-Carter model with only one principal component. The results for the accuracy of the model are similar as with the Lee-Carter model with only one principal component (mostly observed values as jump-off rates are most optimal). Again, we see less differences in outcomes between the different choices of the jump-off rates.

**Table 4:** Mean absolute forecast error (MAFE) of remaining life expectancy at age 65 for the first and fifth year in the forecasting period and standard deviation (SD) of the increase/decrease of the life expectancy at age 65 in 2020 between ten successive forecasts, for six different choices of the jump-off rates applied to a Lee-Carter model with three principal components, for eight Western European countries, men and women combined, fitting periods 1960–2005, 1960–2006, …, 1960–2014

Jump-off rates	FRA	ESP	SWE	BEL	UK	NOR	FIN	NLD	Av^1^
Standard deviation in 2020									
Model values	**0.17**	**0.23**	*0.25*	0.14	0.16	*0.28*	0.50	**0.20**	*0.24*
Last observed values	*0.21*	*0.30*	**0.17**	*0.20*	0.14	0.19	**0.45**	*0.25*	0.24
Average 2 years observed	0.20	0.25	0.18	0.15	**0.13**	**0.17**	0.51	0.20	**0.22**
Average 3 years observed	0.18	0.24	0.19	0.14	0.16	0.22	*0.54*	0.21	0.23
Average 4 years observed	0.18	0.23	0.20	0.13	0.17	0.23	0.53	0.21	0.23
Average 5 years observed	0.18	0.24	0.21	**0.13**	*0.17*	0.24	0.52	0.21	0.24
Mean absolute forecast error in the first year of the forecasting period									
Model values	*0.24*	*0.24*	*0.22*	0.12	*0.21*	*0.22*	*0.36*	*0.21*	*0.23*
Last observed values	**0.14**	0.18	0.13	*0.14*	**0.14**	0.13	**0.30**	0.18	0.17
Average 2 years observed	0.15	0.15	0.12	0.12	0.15	**0.13**	0.31	**0.16**	**0.16**
Average 3 years observed	0.15	**0.15**	0.12	**0.12**	0.17	0.14	0.31	0.17	0.17
Average 4 years observed	0.16	0.16	**0.12**	0.12	0.18	0.15	0.31	0.18	0.17
Average 5 years observed	0.17	0.16	0.13	0.12	0.19	0.15	0.32	0.19	0.18
Mean absolute forecast error in the fifth year of the forecasting period									
Model values	*0.31*	*0.40*	*0.31*	0.18	0.51	*0.43*	*0.38*	*0.41*	*0.37*
Last observed values	**0.18**	**0.23**	**0.15**	**0.17**	**0.43**	0.28	**0.24**	0.36	**0.26**
Average 2 years observed	0.20	0.24	0.20	0.20	0.46	**0.28**	0.25	**0.36**	0.27
Average 3 years observed	0.21	0.25	0.19	0.21	0.48	0.29	0.25	0.37	0.28
Average 4 years observed	0.23	0.27	0.20	*0.22*	0.50	0.30	0.25	0.37	0.29
Average 5 years observed	0.24	0.28	0.22	0.21	*0.51*	0.32	0.28	0.39	0.31

The lowest MAFE is marked in bold, the highest MAFE in italics

^1^Unweigthed average of all eight countries

**Table 5:** Mean absolute (percent) error over the period 1960–2014 and mean error over the period 2005–2014 of the log death rates limited to age 65 and above of the Lee-Carter forecast with three principal components estimated over the period 1960–2014, for eight Western European countries, men and women combined

	FRA	ESP	SWE	BEL	UK	NOR	FIN	NLD
Mean absolute error (1960–2014)	0.020	0.025	0.028	0.030	0.022	0.035	0.042	0.024
Mean absolute percent error (1960–2014)	0.91	1.17	1.35	1.50	1.04	1.54	2.19	1.17
Mean error (2005–2014)	− 0.007	− 0.006	− 0.012	− 0.004	− 0.004	− 0.011	− 0.019	− 0.001

## Appendix 3

In Tables 6 and 7, the results of the different choices for the jump-off rates with the Cairns-Black-Dowd (CBD) model (Cairns et al. 2006), a variant of the Lee-Carter model which relies on the linearity of the logit of 1-year death probabilities at older ages, are presented. With this model, the differences between the outcome of the different choices of the jump-off rates are much more smaller compared to the differences in outcomes for the Lee-Carter model. The fit of this model over the whole fitting period is similar to the fit of the Lee-Carter method, but the mean error in recent years of the fitting period is now positive, which might influence the outcomes as well.

**Table 6:** Mean absolute forecast error (MAFE) of remaining life expectancy at age 65 for the first and fifth year in the forecasting period and standard deviation (SD) of the increase/decrease of the life expectancy at age 65 in 2020 between ten successive forecasts, for six different choices of the jump-off rates applied to the Cairns-Black-Dowd method (ages 65 to 95), for eight Western European countries, men and women combined, fitting periods 1960–2005, 1960–2006, …, 1960–2014

Jump-off rates	FRA	ESP	SWE	BEL	UK	NOR	FIN	NLD	Av^1^
Standard deviation in 2020									
Model values	*0.24*	*0.29*	*0.12*	*0.24*	*0.20*	*0.15*	*0.13*	*0.16*	*0.19*
Last observed values	0.22	0.28	0.11	0.23	0.19	0.15	**0.12**	0.15	0.18
Average 2 years observed	0.22	0.28	0.11	0.23	0.19	0.15	0.13	**0.15**	0.18
Average 3 years observed	0.22	0.28	**0.11**	**0.22**	0.19	0.15	0.13	0.15	0.18
Average 4 years observed	0.22	**0.28**	0.11	0.22	0.19	0.15	0.13	0.15	**0.18**
Average 5 years observed	**0.22**	0.28	0.11	0.22	**0.19**	**0.15**	0.13	0.15	0.18
Mean absolute forecast error in the first year of the forecasting period									
Model values	*0.15*	0.17	**0.08**	*0.15*	*0.12*	**0.11**	*0.12*	**0.11**	*0.13*
Last observed values	**0.13**	*0.17*	0.08	0.14	0.12	0.12	**0.10**	0.13	**0.12**
Average 2 years observed	0.13	0.17	0.08	0.13	0.12	0.12	0.10	0.13	0.12
Average 3 years observed	0.13	0.17	0.08	0.13	0.12	0.12	0.10	0.13	0.12
Average 4 years observed	0.13	0.17	0.09	0.13	0.12	0.12	0.10	0.13	0.12
Average 5 years observed	0.13	**0.17**	*0.09*	**0.13**	**0.12**	*0.12*	0.10	*0.13*	0.12
Mean absolute forecast error in the fifth year of the forecasting period									
Model values	*0.19*	**0.25**	**0.04**	*0.17*	**0.34**	**0.11**	*0.26*	**0.27**	**0.20**
Last observed values	**0.16**	*0.32*	0.06	0.17	*0.35*	0.18	0.19	*0.28*	*0.21*
Average 2 years observed	0.16	0.32	0.06	0.17	0.35	0.18	**0.19**	0.28	0.21
Average 3 years observed	0.16	0.32	*0.06*	**0.17**	0.35	0.18	0.19	0.28	0.21
Average 4 years observed	0.16	0.31	0.06	0.17	0.35	0.19	0.19	0.28	0.21
Average 5 years observed	0.17	0.31	0.06	0.17	0.35	*0.19*	0.19	0.28	0.21

The lowest SD/MAFE is marked in bold, the highest SD/MAFE in italics

^1^Unweigthed average of all eight countries

**Table 7:** Mean absolute (percent) error over the period 1960–2014 and mean error over the period 2005–2014 of the log death rates limited to age 65 and above of the Cairns-Black-Dowd method (ages 65 and above) estimated over the period 1960–2014, men and women combined

	FRA	ESP	SWE	BEL	UK	NOR	FIN	NLD
Mean absolute error (1960–2014)	0.055	0.039	0.041	0.042	0.033	0.042	0.047	0.038
Mean absolute percent error (1960–2014)	2.18	1.71	1.84	2.03	1.65	1.94	2.44	1.89
Mean error (2005–2014)	0.025	0.019	0.016	0.022	0.009	0.013	0.019	0.012

## Appendix 4

**Table 8:** Mean absolute forecast error (MAFE) of remaining life expectancy at age 65 for the first and fifth year in the forecasting period, and standard deviation (SD) of the increase/decrease of the life expectancy at age 65 in 2020 between ten successive forecasts, for six different choices of the jump-off rates applied to a Lee-Carter model, for eight countries, men (a) and women (b), fitting periods 1960–2005, 1960–2006, …, 1960–2014

Jump-off rates	FRA	ESP	SWE	BEL	UK	NOR	FIN	NLD	Av^1^
a. Men									
Standard deviation in 2020									
Model values	0.11	0.11	*0.36*	0.16	*0.16*	*0.26*	*0.52*	0.13	*0.23*
Last observed values	*0.15*	*0.23*	0.10	*0.21*	0.13	0.16	**0.13**	*0.14*	0.16
Average 2 years observed	0.09	0.08	0.09	0.11	0.09	0.11	0.14	0.09	0.10
Average 3 years observed	0.08	0.08	**0.07**	0.08	0.08	**0.09**	0.15	0.08	0.09
Average 4 years observed	0.06	**0.05**	0.08	0.07	0.07	0.11	0.15	0.07	0.08
Average 5 years observed	**0.05**	0.05	0.09	**0.06**	**0.06**	0.10	0.14	**0.06**	**0.08**
Mean absolute forecast error in the first year of the forecasting period									
Model values	**0.11**	**0.14**	*0.69*	*0.52*	*0.75*	*0.80*	*0.58*	*1.15*	*0.59*
Last observed values	0.12	0.18	**0.10**	**0.16**	**0.12**	**0.16**	0.10	**0.20**	**0.14**
Average 2 years observed	0.13	0.17	0.13	0.17	0.16	0.17	0.10	0.25	0.16
Average 3 years observed	0.15	0.19	0.17	0.19	0.21	0.20	**0.09**	0.34	0.19
Average 4 years observed	0.19	0.22	0.21	0.22	0.27	0.26	0.12	0.44	0.24
Average 5 years observed	*0.23*	*0.24*	0.25	0.26	0.34	0.32	0.15	0.53	0.29
Mean absolute forecast error in the fifth year of the forecasting period									
Model values	**0.21**	**0.35**	*0.93*	*0.69*	*1.04*	*1.25*	*0.84*	*1.60*	*0.86*
Last observed values	0.25	0.42	**0.35**	**0.24**	**0.42**	**0.48**	**0.16**	**0.65**	**0.37**
Average 2 years observed	0.28	0.45	0.38	0.28	0.45	0.53	0.17	0.74	0.41
Average 3 years observed	0.33	0.50	0.42	0.33	0.50	0.58	0.20	0.84	0.46
Average 4 years observed	0.38	0.54	0.47	0.39	0.56	0.65	0.26	0.95	0.52
Average 5 years observed	*0.43*	*0.57*	0.52	0.44	0.62	0.73	0.31	1.05	0.58
b. Women									
Standard deviation in 2020									
Model values	0.13	0.14	*0.15*	*0.23*	0.18	*0.40*	*0.62*	*0.26*	*0.26*
Last observed values	*0.21*	*0.24*	0.14	0.19	*0.20*	0.19	0.22	0.15	0.19
Average 2 years observed	0.13	0.11	0.08	0.11	0.12	0.12	**0.18**	**0.09**	0.12
Average 3 years observed	0.11	0.09	0.04	0.09	0.11	0.10	0.23	0.09	0.11
Average 4 years observed	0.10	**0.05**	0.03	**0.06**	0.08	0.09	0.21	0.09	0.09
Average 5 years observed	**0.08**	0.06	**0.03**	0.07	**0.06**	**0.07**	0.20	0.09	**0.08**
Mean absolute forecast error in the first year of the forecasting period									
Model values	**0.13**	*0.20*	**0.08**	*0.14*	*0.47*	*0.14*	*0.54*	0.15	*0.23*
Last observed values	0.14	0.17	*0.11*	**0.12**	**0.14**	0.13	0.11	0.12	0.13
Average 2 years observed	0.15	**0.15**	0.10	0.13	0.17	0.11	**0.09**	**0.10**	**0.13**
Average 3 years observed	0.15	0.15	0.09	0.13	0.19	0.10	0.13	0.14	0.14
Average 4 years observed	*0.17*	0.16	0.10	0.13	0.21	0.10	0.17	0.17	0.15
Average 5 years observed	0.16	0.17	0.08	0.13	0.24	**0.09**	0.20	*0.20*	0.16
Mean absolute forecast error in the fifth year of the forecasting period									
Model values	*0.26*	*0.37*	*0.12*	*0.25*	*0.61*	0.14	*0.46*	0.24	*0.31*
Last observed values	**0.18**	**0.26**	**0.09**	**0.17**	**0.36**	**0.11**	**0.25**	**0.21**	**0.20**
Average 2 years observed	0.21	0.28	0.10	0.18	0.38	0.12	0.26	0.23	0.22
Average 3 years observed	0.19	0.31	0.11	0.18	0.40	0.11	0.27	0.27	0.23
Average 4 years observed	0.22	0.33	0.10	0.20	0.41	0.15	0.28	0.31	0.25
Average 5 years observed	0.22	0.34	0.10	0.19	0.44	*0.16*	0.29	*0.34*	0.26

The lowest SD/MAFE is marked in bold, the highest SD/MAFE in italics

^1^Unweigthed average of all eight countries

**Table 9:** Mean absolute (percent) error over the period 1960–2014 and mean error over the period 2005–2014 of the log death rates limited to age 65 and above of the Lee-Carter forecast estimated over the period 1960–2014, men (a) and women (b)

	FRA	ESP	SWE	BEL	UK	NOR	FIN	NLD
a. Men								
Mean absolute error (1960–2014)	0.027	0.039	0.046	0.049	0.043	0.061	0.064	0.067
Mean absolute percent error (1960–2014)	1.39	1.91	2.25	2.61	2.06	2.82	3.66	3.06
Mean error (2005–2014)	0.005	0.019	− 0.046	− 0.028	− 0.047	− 0.052	− 0.045	− 0.063
b. Women								
Mean absolute error (1960–2014)	0.029	0.042	0.036	0.039	0.035	0.046	0.062	0.040
Mean absolute percent error (1960–2014)	1.09	1.79	1.57	1.73	1.36	1.89	2.82	1.73
Mean error (2005–2014)	0.018	− 0.007	0.004	0.015	− 0.034	0.000	− 0.055	0.010

## Appendix 5

In Tables 10 and 11, the results of the different choices for the jump-off rates with the Lee-Carter model are presented for the life expectancy at birth (eo). For the most accurate forecast, the last observed values as jump-off rates are the best choice, similar as with the life expectancy at age 65 (e65). For Spain, the best option of the model values as jump-off rates is more clear than in the results with e65. For France, an average of multiple years as jump-off rates is better than the last observed values, but the difference is small. For the most robust forecast, an average of multiple years is the best choice for the jump-off rates based on the results of e0, which was also the best choice based on the results of e65.

**Table 10:** Mean absolute forecast error (MAFE) of remaining life expectancy at birth for the first and fifth year in the forecasting period, and standard deviation (SD) of the increase/decrease of the life expectancy at birth in 2020 between ten successive forecasts, for six different choices of the jump-off rates applied to a Lee-Carter model, for eight countries, men and women combined, fitting periods 1960–2005, 1960–2006, …, 1960–2014

Jump-off rates	FRA	ESP	SWE	BEL	UK	NOR	FIN	NLD	Av^1^
Standard deviation in 2020									
Model values	0.16	0.15	*0.36*	*0.25*	*0.22*	*0.37*	*0.71*	*0.25*	*0.31*
Last observed values	*0.20*	*0.25*	0.09	0.22	0.18	0.11	0.25	0.16	0.18
Average 2 years observed	0.12	0.11	0.08	0.11	0.12	**0.10**	0.19	0.11	0.12
Average 3 years observed	0.11	0.10	0.07	0.10	0.10	0.11	0.20	0.11	0.11
Average 4 years observed	0.10	**0.05**	**0.07**	0.07	0.08	0.13	0.20	0.11	0.10
Average 5 years observed	**0.08**	0.07	0.09	**0.07**	**0.06**	0.12	**0.19**	**0.10**	**0.10**
Mean absolute forecast error in the first year of the forecasting period									
Model values	*0.21*	**0.17**	*0.38*	**0.08**	*0.46*	*0.38*	*0.42*	*0.47*	*0.32*
Last observed values	0.15	0.19	**0.08**	0.15	**0.13**	**0.11**	0.13	**0.16**	**0.14**
Average 2 years observed	0.14	0.19	0.08	0.15	0.15	0.12	**0.09**	0.20	0.14
Average 3 years observed	**0.14**	0.21	0.10	0.15	0.19	0.16	0.10	0.26	0.16
Average 4 years observed	0.15	0.24	0.11	0.16	0.24	0.22	0.12	0.33	0.20
Average 5 years observed	0.19	*0.27*	0.13	*0.17*	0.29	0.27	0.13	0.42	0.23
Mean absolute forecast error in the fifth year of the forecasting period									
Model values	0.24	**0.37**	*0.49*	0.18	*0.78*	*0.74*	*0.62*	0.70	*0.51*
Last observed values	0.17	0.48	**0.16**	**0.16**	**0.50**	**0.38**	**0.25**	**0.39**	**0.31**
Average 2 years observed	**0.16**	0.52	0.16	0.17	0.51	0.40	0.25	0.44	0.33
Average 3 years observed	0.18	0.58	0.19	0.19	0.52	0.45	0.26	0.52	0.36
Average 4 years observed	0.22	0.61	0.22	0.21	0.54	0.52	0.28	0.62	0.40
Average 5 years observed	*0.25*	*0.65*	0.25	*0.24*	0.57	0.60	0.31	*0.71*	0.45

The lowest SD/MAFE is marked in bold, the highest SD/MAFE in italics

^1^Unweighted average of all countries

**Table 11:** Mean absolute (percent) error over the period 1960–2014 and mean error over the period 2005–2014 of the log death rates of the Lee-Carter forecast estimated over the period 1960–2014, men and women combined

	FRA	ESP	SWE	BEL	UK	NOR	FIN	NLD
Mean absolute error (1960–2014)	0.054	0.076	0.087	0.071	0.051	0.097	0.094	0.060
Mean absolute percent error (1960–2014)	1.07	1.49	1.53	1.39	1.08	1.70	1.88	1.22
Mean error (2005–2014)	− 0.005	− 0.010	− 0.010	− 0.010	− 0.007	− 0.016	− 0.010	− 0.015

## Appendix 6

In Figure 3, an example of a forecast using interpolation is shown for the Netherlands. It starts with a forecast using a jump-off rate that gives the most accurate forecast in the first year (red line). Subsequently, a forecast using jump-off rates that is most robust in the fifth year of the forecast period is made (green line). Between the two forecast, each year, more weight is given to the most robust forecast until the weight is equal to 1 from the fifth year of the forecast onwards (dashed purple line).

## Abbreviations

BEL: Belgium
e65: Life expectancy at age 65
ESP: Spain
FIN: Finland
FRA: France
MAE: Mean absolute error
NLD: the Netherlands
NOR: Norway
SD: Standard deviation
SWE: Sweden
UK: United Kingdom
